# Foreign Body in the Breast: A Case Report

**DOI:** 10.7759/cureus.73162

**Published:** 2024-11-06

**Authors:** Hakan Baysal, Tunc Eren, Gulnur Erdem, Celal Karatas, Begumhan Baysal, Orhan Alimoglu

**Affiliations:** 1 General Surgery, Istanbul Medeniyet University Göztepe Prof. Dr. Süleyman Yalçın Şehir Hastanesi, Istanbul, TUR; 2 Radiology, Istanbul Medeniyet University, Istanbul, TUR

**Keywords:** breast, foreign bodies, mammography, radiology, surgery

## Abstract

Foreign bodies in the breast are rarely encountered and may infrequently lead to complications such as persistent breast pain, abscess, and/or granuloma formation, pneumothorax, or cardiac tamponade. We aimed to present a case with a foreign body in her right breast, which was diagnostically missed in previous screenings. A 44-year-old asymptomatic woman, who was included in the national breast cancer screening program, was found to have a 2 cm-sized metallic foreign body in the upper-outer quadrant of her right breast via mammographic imaging. Retrospective clinical evaluations revealed that the foreign body had been present in her breast for at least five years. She was successfully treated via wire-assisted surgery. Although they may remain asymptomatic for long periods, foreign bodies detected in the breast are suggested to be surgically removed due to the possibility of considerable subsequent local or thoracic complications.

## Introduction

Foreign bodies in the breast may rarely be encountered in surgical outpatient clinics. The material and localization of the foreign body encountered and the methods to be applied for the treatment of the patient are important. The most commonly encountered foreign bodies in the breast may be surgical clips or wire hook pieces retained from excisional biopsies [1]. Other reported substances include metallic and plastic bodies, lead cores, and glass fragments [1,2]. Foreign bodies may cause breast pain due to abscesses or granuloma formation. Additionally, migration of a foreign body may lead to rare complications such as cardiac tamponade and pneumothorax [3]. Otherwise, most cases are asymptomatic [3]. Mammography (MG) is one of the imaging methods that can be used to identify foreign bodies in the breast. Invasive or minimally invasive procedures have been suggested for the removal of foreign bodies detected in the breast [1,2,4]. In this case report, we aimed to present the surgical excision of a foreign body detected in the right breast on screening MG by wire localization.

## Case presentation

**Figure 1:**
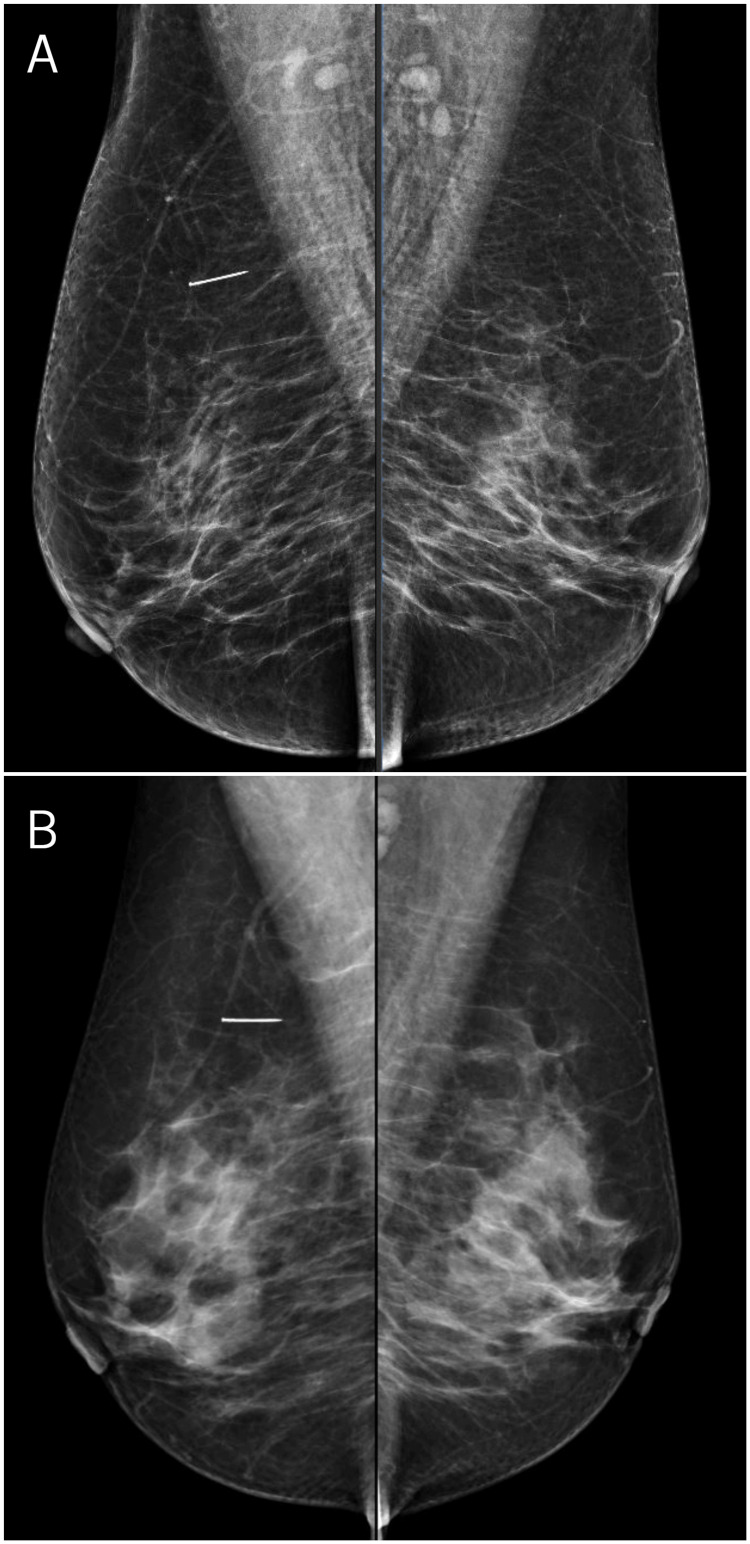
Mammography. (A) Mediolateral-oblique radiographic image from preoperative mammography in 2023. (B) Mediolateral-oblique radiographic image from mammography performed five years earlier in 2018.

A 44-year-old woman, who was included in the national breast cancer screening program at the age of 40 and was diagnosed with a nodular lesion in the upper outer quadrant of her left breast defined as category 2 according to the Breast Imaging-Reporting and Data System (BI-RADS), was admitted to the Department of General Surgery without having attended any follow-ups in the past five years. She was asymptomatic at presentation, and her breast examination was unremarkable. Her routine laboratory test results were within normal ranges. A current MG performed for screening purposes revealed a 2 cm-sized metallic foreign body (MFB) in the upper-outer quadrant of her right breast (Figure 1). When her previous MG, which had been performed at a different center five years earlier, was reviewed retrospectively, the aforementioned 2 cm MFB was found to be present in the upper-outer quadrant of the right mediolateral-oblique (MLO) radiograph as well; however, it had not been reported in the MG report at that time (Figure 1). Therefore, the patient had no prior knowledge of the foreign body in her breast.

**Figure 2:**
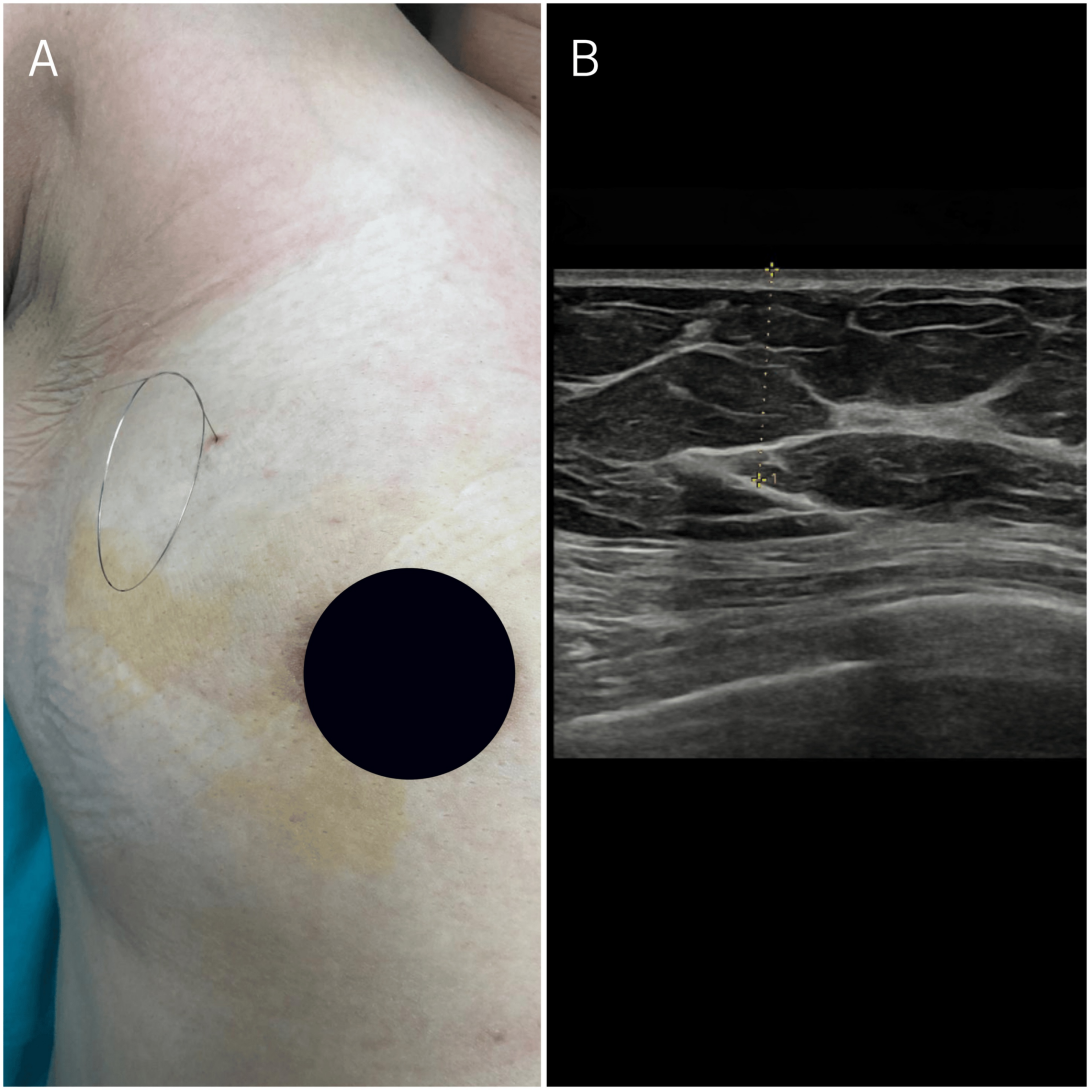
Interventional radiology. (A) The breast prepared for surgical excision after wire localization of the foreign body. (B) Ultrasound image of the foreign body prior to wire localization.

The case was evaluated at the Surgery-Radiology Multidisciplinary Board meeting, and the operation was decided upon at the patient's request. The wire localization method was carried out in the Department of Radiology to provide intraoperative guidance for the operating surgeon (Figure 2). The MFB, which was found at a depth of 3 cm from the skin extending in an oblique fashion within the upper-outer quadrant of the right breast, was marked with a 20G/10 cm needle wire system under ultrasound (US) guidance by advancing approximately 3 cm perpendicularly at a 90° angle (Figure 2).

**Figure 3:**
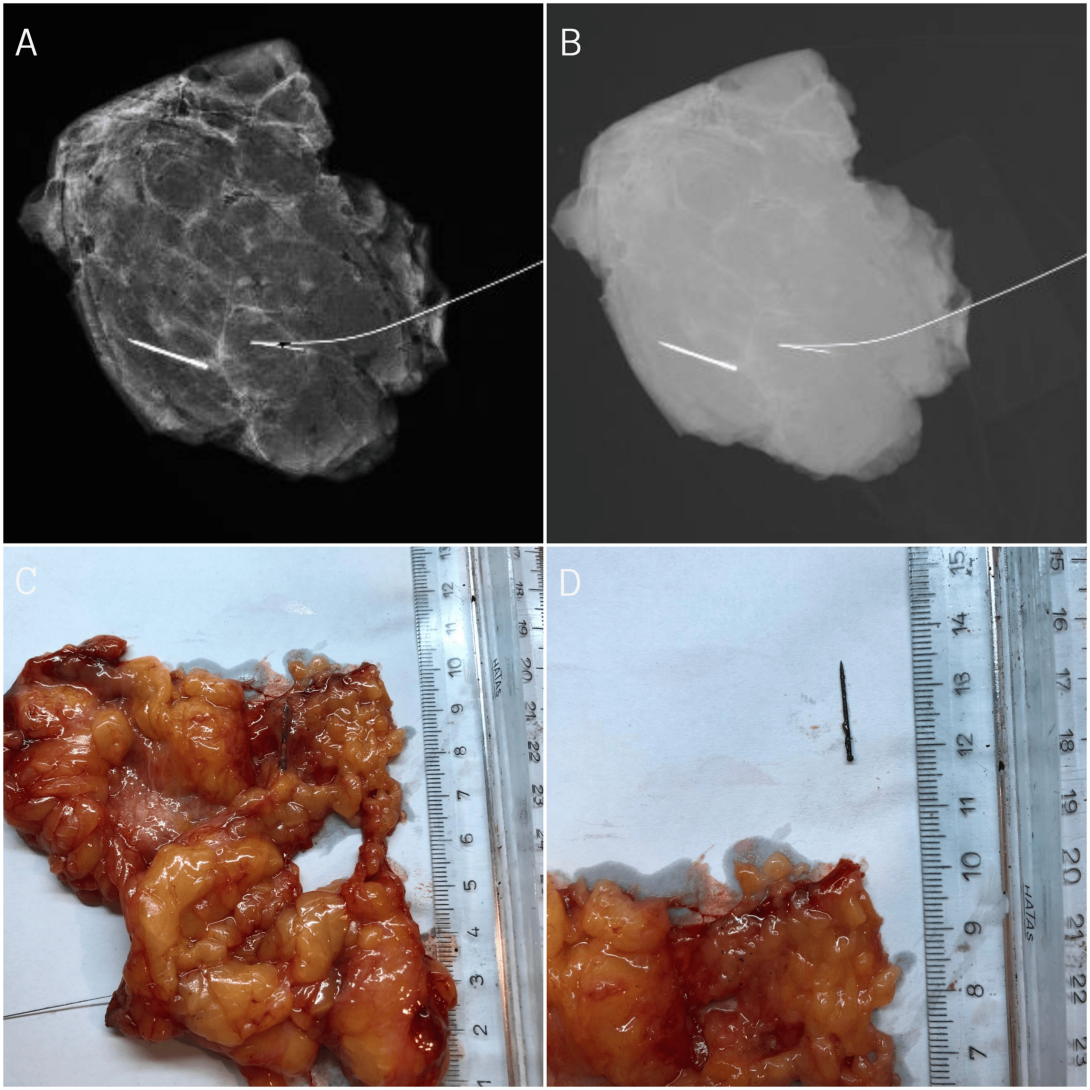
Surgery. (A) Processed radiographic image of the wire-localized specimen containing the foreign body. (B) Raw radiographic image of the wire-localized specimen containing the foreign body. (C) Excised specimen. (D) Foreign body extracted from the removed specimen.

Following the radiologic intervention, the patient was transferred to the operating room, and the MFB was removed along with minimal tissue resection through a 3 cm surgical incision created in the upper-outer quadrant of her right breast, followed by a specimen radiography performed intraoperatively to confirm that the excised tissue contained the foreign body (Figure 3).

She was discharged uneventfully on the postoperative first day and reported no new symptoms at follow-ups during her first postoperative year.

## Discussion

Foreign bodies in the breast are rare, and there are only a limited number of reported cases. They typically consist of a wide variety of substances such as metal, glass particles, plastic, paraffin, or silicone [5]. Often, MFBs or particles, most commonly retained after gunshot wounds and interventional radiological or surgical procedures, consist of fragments of suture needles, surgical materials, and clips or localization wires. It has been reported that the most common types of MFBs in the breast are broken pieces of guide-wire and surgical clips [6]. Although MFBs in the breast can cause local inflammation findings such as pain, abscesses, and/or granuloma formation, they may also present with more serious conditions such as pneumothorax or cardiac tamponade [7]. Most foreign bodies in the breast are detected on MG or breast US. Since echogenic foci on US can include differential diagnoses such as gas, calcifications, fat necrosis, silicone, and metals, MG is recommended as the imaging modality of choice to allow for a more specific diagnosis. Many cases may remain asymptomatic for a long period before diagnosis is made, as in our case. Depending on their composition and potential harm, not all foreign bodies in the breast need to be removed [8]. However, removal may be considered if there is severe pain, a risk of future harm to the patient, or if desired by the patient. Our case was asymptomatic, and the removal of the foreign body was carried out upon the patient's request. In the patient’s history, it was noted that she had fastened her headscarf with metallic pins, and it was suspected that a foreign body might have entered the breast possibly in connection with the patient’s clothing action, although the patient did not remember the penetration of the foreign body into the breast. The options for foreign body removal from the breast usually depend on the type, size, and location of the foreign body. Palpable lesions are usually simple to remove. For non-palpable MFBs, removal may be carried out via mammotome biopsy, or via surgery under the guidance of either a guide-wire or fluoroscopy [6,9,10]. The radioguided occult lesion localization (ROLL) technique has also been reported as another option [2,9]. However, the ROLL technique has some disadvantages such as the potential for injection of the radionuclide into the wrong site, a ductography-like appearance due to intraductal injection of the radionuclide, contamination of the skin with the radionuclide, and the necessity of using a gamma-probe [2]. Wire localization, which we used in our case, offered shorter hospitalization and operation times, smaller incisions, good aesthetic results compared to other techniques, and was cost-effective [1,2,4]. We consider the clinical features of the presented case, being detected only by screening MG, being associated with clothing and treated via wire-assisted removal with very little tissue loss due to its intramammary depth, to validate it as an interesting case.

## Conclusions

A 44-year-old woman with a foreign body in her right breast that remained undetected for five years was successfully treated via wire-assisted surgical removal, as presented herein. A foreign body in the breast, even one harbored for long periods and remaining asymptomatic, may eventually lead to late complications, including abscess and/or granuloma formation, pneumothorax, or cardiac tamponade. Therefore, we recommend surgery as the preferred treatment approach for patients diagnosed with a foreign body in the breast, particularly when there is a potential complication risk associated with the foreign body or if surgical removal is desired by the patient.

## References

[1] Scaperrotta GP, Capalbo E, Cartia F, Ferranti C, Vigano S, Panizza P (2015). Breast foreign body extraction using the breast lesion excision system. J Vasc Interv Radiol.

[2] Aydogan F, Atasoy D, Olgun DC, Dikici AS, Aliyev A, Gazioglu E (2010). Extraction of a foreign body from the breast parenchyma using radioguided occult lesion localisation (ROLL) technique: a new approach. Br J Radiol.

[3] Vesna D, Tatjana A, Slobodan S, Slobodan N (2004). Cardiac tamponade caused by migration of a swallowed sewing needle. Forensic Sci Int.

[4] Sibilio A, Bucchi E, Alfieri C, Marongiu F, Curcio A (2022). Successful retrieval of a needle point from the breast through a vacuum-assisted breast biopsy system. Acta Radiol Open.

[5] Monib S, Anis K (2021). Iatrogenic breast foreign body seen on a screening mammogram. Indian J Surg.

[6] Montrey JS, Levy JA, Brenner RJ (1996). Wire fragments after needle localization. AJR Am J Roentgenol.

[7] Joret MO, El-Haddawi F (2021). Intraperitoneal migration of a hookwire following wide local excision of a breast lesion presenting as a spontaneous pneumothorax. BMJ Case Rep.

[8] Khanna A, Peters MS, Glazebrook KN, Nguyen GH, Tsetse CM, Sae-Kho TM (2024). Glass shards masquerading as calcifications in the breast: a case report. Radiol Case Rep.

[9] Dal F, Ökmen H, Yılmaz MK, Sarı S, Nazlı MA, Arslan E (2017). Extraction of a foreign body from the breast using radio-guided occult lesion localization (roll): metallic foreign body in the breast. Eur J Breast Health.

[10] Parker SH, Kercher JM, Dennis MA (1999). Sonographically guided mammotome extraction of retained localization wire. AJR Am J Roentgenol.

